# Alternative Splicing Analysis Revealed That the Transcription Factor PacC Shapes the Virulence of the Dermatophyte *Trichophyton interdigitale*

**DOI:** 10.3390/ijms27062634

**Published:** 2026-03-13

**Authors:** Mayara I. G. Azevedo, João Neves-da-Rocha, Pablo R. Sanches, Vanderci M. Oliveira, Nilce M. Martinez-Rossi, Antonio Rossi

**Affiliations:** Department of Genetics, Ribeirão Preto Medical School, University of São Paulo (USP), Ribeirão Preto 14049-900, SP, Brazil; itala11@gmail.com (M.I.G.A.); joao.neves.fonseca@usp.br (J.N.-d.-R.); psanches@usp.br (P.R.S.); cuca@fmrp.usp.br (V.M.O.); anrossi@usp.br (A.R.)

**Keywords:** intron retention, exon skipping, fungal virulence, epigenetic regulation, ergosterol

## Abstract

Rapid responses to environmental changes are essential for maintaining fitness. In pathogenic fungi such as the dermatophyte *Trichophyton interdigitale*, appropriate responses to environmental shifts determine successful infection. Transcriptional regulation and alternative splicing (AS) are key modulators of fungal adaptation and pathogenesis. Here, we validated the role of the transcription factor PacC in coordinating AS in *T. interdigitale* grown in infection-mimicking medium. RNA-seq analysis of a *ΔpacC* mutant revealed a predominance of intron retention events, mainly involving introns 1 and 2, indicating defective splicing and potential nonsense-mediated decay of genes related to ion transport, metabolism, and genome maintenance. These alterations compromised energy balance, ergosterol biosynthesis, and cellular homeostasis. PacC-dependent AS generated alternative isoforms of cytoskeletal and metabolic proteins, including myosin-1 and a GH3 β-glucosidase, potentially modulating enzymatic activity, metabolic burden, and cell wall remodeling during infection. Exon-skipping in the chromatin remodeler RSC1 suggests PacC involvement in epigenetic regulation under host-mimicking conditions. Transmission electron microscopy revealed possible Woronin bodies, cytoplasmic disruption, and cell wall thinning in the mutant. Overall, PacC integrates transcriptional and post-transcriptional regulation to promote adaptation, survival, and virulence, highlighting AS as a regulatory layer linking environmental sensing to metabolic and epigenetic plasticity in pathogenic fungi.

## 1. Introduction

Fungi display wide ranging lifestyles, inhabit diverse ecological niches, and can adapt to the accompanying environmental changes [1]. The ability to detect and respond to different environmental conditions is critical for survival and pathogenicity [2]. Among these environmental factors, pH plays a central role in modulating fungal growth, development, and host invasion as many pathogens actively acidify or alkalinize their microenvironment to optimize enzymatic activity, growth, and blunt host defenses [3,4,5]. To adapt to different pH levels, transcription factors (TFs) alter gene expression, leading to genetic and metabolic reprogramming [6].

PacC is a TF in the PacC/Pal signal transduction pathway that controls responses to environmental pH fluctuations [7]. PacC is linked to growth, germination, pathogenicity, and biosynthesis of secondary metabolites, and it regulates genes involved in morphological changes, cell wall biosynthesis, virulence factors, and stress responses [8,9,10]. PacC balances responses to nutrient availability and stress by functioning as both an activator and a repressor of transcription, making it crucial for adaptation and virulence [11].

Another key factor in environmental adaptation is post-transcriptional regulation of gene expression via alternative splicing (AS). AS generates multiple mRNA isoforms from a single gene by selectively including introns or excluding specific exons, thereby enhancing transcriptomic and proteomic complexity [12,13]. AS plays a crucial role in the production of alternative protein isoforms that contribute to fungal pathogenicity, drug resistance, and environmental adaptation [14,15,16]. This process can be coupled with nonsense-mediated RNA decay (NMD), which controls the levels of productive mRNA isoforms [17]. However, disturbances in splicing machinery or regulatory mechanisms can produce aberrantly spliced transcripts, thereby disrupting the fine-tuned regulation of gene expression [15,18]. A clear example is the transcription factor StuA, which modulates AS and influences the MAPK signaling pathway [19]. Given that transcription factors, such as StuA, can modulate splicing, it is possible that PacC influences this process, particularly during fungal adaptation to the host environment [20].

Dermatophytes are keratinophilic fungi; their interaction with host tissue triggers several metabolic adaptations, including secretion of proteases that degrade keratin and activation of energy pathways such as glycolysis, the tricarboxylic acid (TCA) cycle, and amino acid degradation [21]. This metabolic shift is mediated by TFs such as StuA and PacC [22,23]. *Trichophyton interdigitale* is an anthropophilic dermatophyte and a primary cause of persistent and recurrent cutaneous mycoses, which are a serious public health burden [24]. A Δ*pacC* mutant of *T. interdigitale* showed decreased growth in both keratin medium and human nails, presenting lower keratinolytic activity than the wild-type strain [25]. Another study using the same Δ*pacC* mutant revealed that PacC regulates the transcription of N- and O-linked mannosyltransferase genes, which can alter the glycosylation profile of fungal proteins and influence their activities and half-lives [26].

Despite its well-known roles in infection and metabolism, the influence of PacC on post-transcriptional regulation via modulation of AS remains poorly explored. Therefore, we investigated whether PacC modulates AS in *T. interdigitale* cultivated in keratin medium, which mimics infection.

## 2. Results

### 2.1. Genome-Wide Alternative Splicing (AS) Profiling of the ΔpacC Mutant

**Figure 1:**
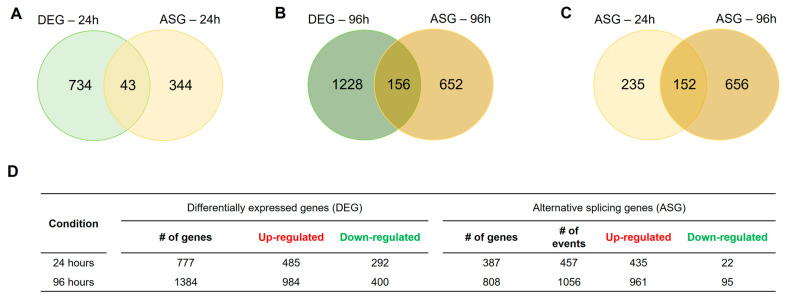
Venn diagrams illustrating the differentially expressed genes (DEGs) and alternatively spliced genes (ASGs) in a *T. interdigitale* Δ*pacC* mutant compared to the wild-type (WT) strain grown in keratin medium for 24 and 96 h. (**A**,**B**) DEGs and ASGs at (**A**) 24 h and (**B**) 96 h. (**C**) ASGs at 24 and 96 h. (**D**) Table summarizing the up- and down-regulated DEGs and ASGs. The symbol # refers to the total number of genes or events.

To simulate infection-like conditions, *T. interdigitale* was grown in keratin medium for 24 and 96 h. To assess the possible role of PacC-mediated AS in successful host invasion, we evaluated the AS profile of the WT and Δ*pacC* mutant. Analysis of the *T. interdigitale* Δ*pacC* mutant and WT transcriptomes revealed 777 differentially expressed genes (DEGs) and 387 alternatively spliced genes (ASGs), with 43 genes exhibiting both differential expression and AS after 24 h of culture (Figure 1A). At 96 h, we detected 1384 DEGs, 808 ASGs, and 156 genes that were both differentially expressed and alternatively spliced (Figure 1B). Notably, 152 genes were alternatively spliced at both time points, indicating that this event was independent of culture time (Figure 1C).

Similar numbers of up-regulated DEGs and ASGs were observed at 24 h: 485 and 435, respectively. However, the number of down-regulated DEGs (292) was more than 13 times higher than the number of ASGs (22). A similar pattern was observed at 96 h, with 984 up-regulated DEGs and 961 up-regulated ASGs, and 400 down-regulated DEGs and 95 down-regulated ASGs (Figure 1D).

**Figure 2:**
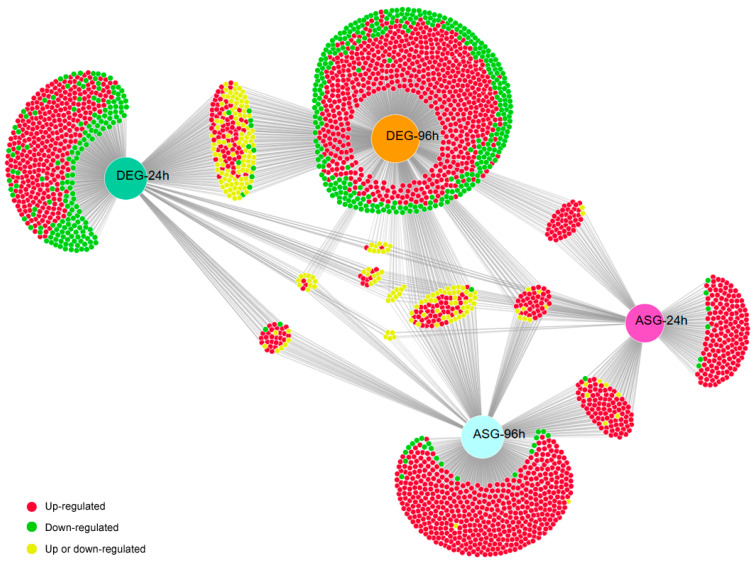
Expression network of differentially expressed genes (DEGs) and alternatively spliced genes (ASGs) in the Δ*pacC* mutant grown in keratin medium.

**Figure 3:**
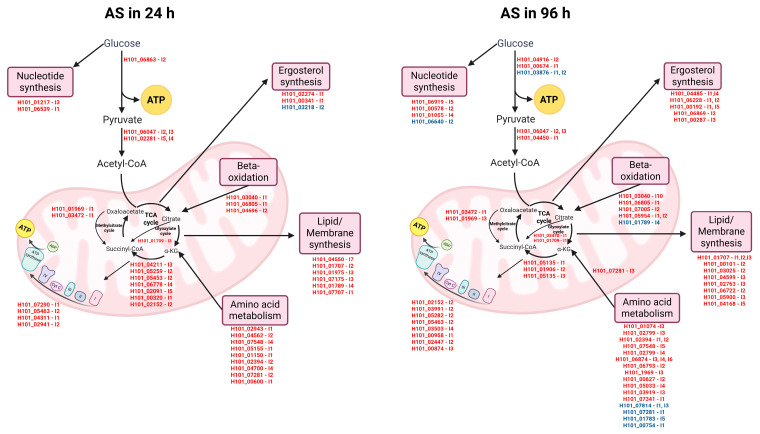
Modulated alternative splicing (AS) events in genes associated with energy metabolism and biosynthesis at 24 h and 96 h in the Δ*pacC* mutant vs. the wild-type strain. The panels shown the major metabolic pathways with altered AS in the mutant, including glycolysis, the TCA cycle, β-oxidation, amino acid metabolism, nucleotide synthesis, ergosterol synthesis, and lipid/membrane biosynthesis. The differential ASGs are shown next to each pathway, with specific intron retention events indicated (I1, I2, I3, etc.). Genes in red represent events with increased relative abundance, and genes in blue represent events with decreased relative abundance. The schematic mitochondrial representation highlights the metabolic interconnections impacted at the two time points. Abbreviations: α-Ketoglutarate (α-KG); adenosine triphosphate (ATP); adenosine diphosphate (ADP); Cytochrome C (Cyt C); Mitochondrial complexes (I, II, III, and IV). Created in BioRender. Rossi, A. (2026) https://www.biorender.com/toujg41 (accessed on 4 February 2026).

Transcriptome analysis revealed a network of simultaneously modulated DEGs and ASGs at 24 and 96 h (Figure 2). The most commonly up-regulated AS events in the mutant at both 24 and 96 h were retention of introns 1 and/or 2 (IR1 and IR2) (Table 1). In comparison, the down-regulated AS events showed an intriguing phenomenon at 24 h: half of the events were exon skipping (Table 2). A closer look at the primary metabolism showed that AS significantly affect glycolysis, the electron transport chain, and key biosynthetic pathways (Figure 3). At both time points, amino acid metabolism and lipid/membrane biosynthesis were greatly affected. However, at 24 h, seven AS events were related to the TCA cycle, whereas at 96 h, this number decreased to three. The opposite was observed for genes related to ergosterol synthesis, with three at 24 h and eight at 96 h. Notably, most AS events were up-regulated and most were IR1 and/or IR2. These analyses showed that the mutant might produce aberrant AS isoforms, since most IR1 and IR2 led to changes in the reading frame and early stop codons, probably activating nonsense-mediated mRNA decay.

**Figure 4:**
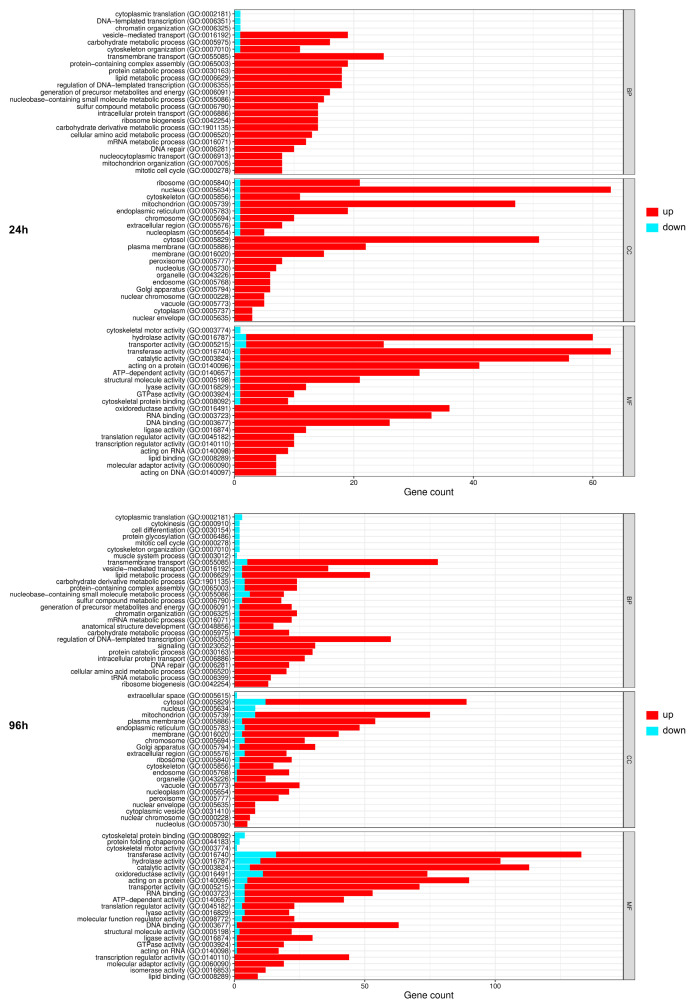
Functional annotation of the most representative genes modulated by Δ*pacC* vs. the wild-type strain of *T. interdigitale* based on Gene Ontology. The green and red bars indicate the down- and up-regulated events, respectively, after 24 h and 96 h in keratin medium. Categories include biological processes (BP), molecular functions (MF), and cellular components (CC).

Functional enrichment analysis of the genes modulated by AS in the Δ*pacC* mutant revealed that, at 24 h, genes encoding transporters and nucleolar proteins were the most commonly affected, and these genes were up-regulated, as were genes encoding hydrolases. In contrast, at 96 h, while the patterns for transporter and hydrolase genes were similar, the number of nucleolar component genes was nearly seven times lower, and these genes were down-regulated. Genes encoding cytosolic proteins were most affected at 96 h (Figure 4). An analysis of the AS events in genes involved in transcriptional regulation and genome maintenance in the Δ*pacC* mutant (Supplementary Table S1) showed that, out of 126 AS events, up-regulation of IR1 and IR2 accounted for 46.3% and 68.2% of the total events at 24 and 96 h, respectively. The analysis also showed that several splicing factors, TFs, and DNA repair proteins undergo AS, suggesting alterations in nuclear metabolism.

### 2.2. Alternative Splicing Validation

**Figure 5:**
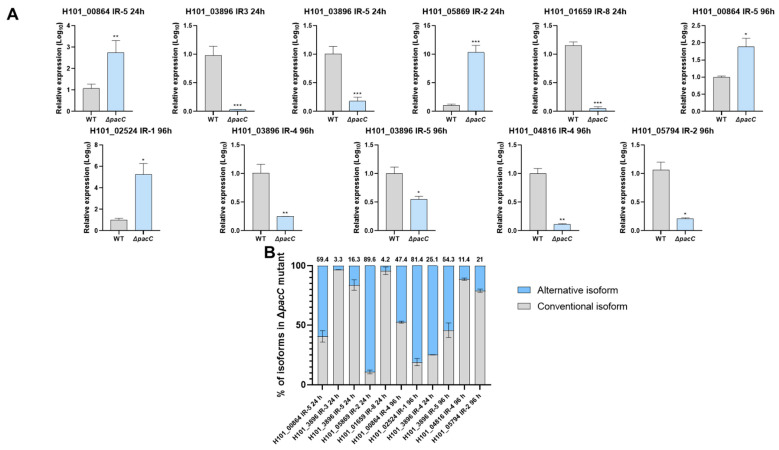
Quantitative polymerase chain reaction validation of the expression levels of several intron retention (IR) events detected in the Δ*pacC* mutant using WT as a reference. (**A**) Expression of selected IR events in the WT and Δ*pacC* strains at 24 h and 96 h. (**B**) Percentage of validated transcripts in the Δ*pacC* mutant grown in a keratin medium. The alternative isoforms (with IR) are shown in blue, and the conventional isoforms are shown in gray. The numbers above each bar are the percentage of AS isoforms. Significance is indicated as follows: * *p* < 0.05, ** *p* < 0.01, and *** *p* < 0.001.

Based on our detailed examination of the AS events and enrichment analysis, we selected candidates for qPCR validation of intron retention (IR) modulation. These included four AS events associated with hydrolases (*H101_03896*, IR3, IR4, and IR5; *H101_04816*, IR4), two related to ATPase activity (*H101_05869*, IR2 and *H101_02524*, IR1), one linked to a myosin-1 protein (*H101_01659*, IR8), one associated with a transmembrane calcium transport protein (*H101_00864*, IR5), and one related to kinase activity (*H101_05794*, IR2) (Figure 5A). Some of the selected events were observed after 24 and 96 h, including down-regulation of IR5 in *H101_03896*, which encodes a β-glucosidase and up-regulation of IR5 in *H101_00864*, which encodes a transmembrane calcium transporter. The results showed that the PacC modulates the percentage of the alternative isoforms, depending on the gene, the intron, and the environmental conditions (Figure 5B).

### 2.3. The ΔpacC Mutant Has Reduced Ergosterol Content

**Figure 6:**
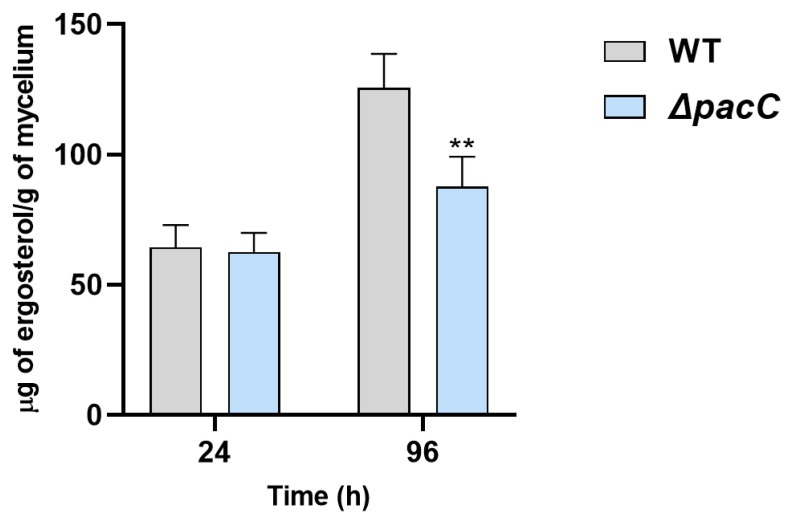
Ergosterol content in the wild-type and Δ*pacC* strains. Quantification of ergosterol (µg/g of mycelium) in the wild-type (WT) and Δ*pacC* strains after 24 and 96 h of growth in keratin medium. Significance is indicated as ** *p* < 0.01.

As stated above, genes in the ergosterol biosynthesis pathway underwent AS in the Δ*pacC* mutant, especially at 96 h, with eight events. Five of the eight events that occurred at 96 h led to an early stop codon and produced dysfunctional isoforms of crucial enzymes in this pathway, such as C-14 sterol reductase (Erg 24) (Supplementary Table S2). To determine whether the AS events related to the ergosterol biosynthesis pathway in the Δ*pacC* mutant impact ergosterol content, we measured and compared ergosterol content in the mutant and WT strains (Figure 6). At 24 h, there was no significant difference between the Δ*pacC* and WT strains. However, at 96 h, the Δ*pacC* mutant contained 30.32% less ergosterol than the WT strain.

### 2.4. Intron Retention in the ΔpacC Mutant Generates Different Isoforms of β-glucosidase

**Figure 7:**
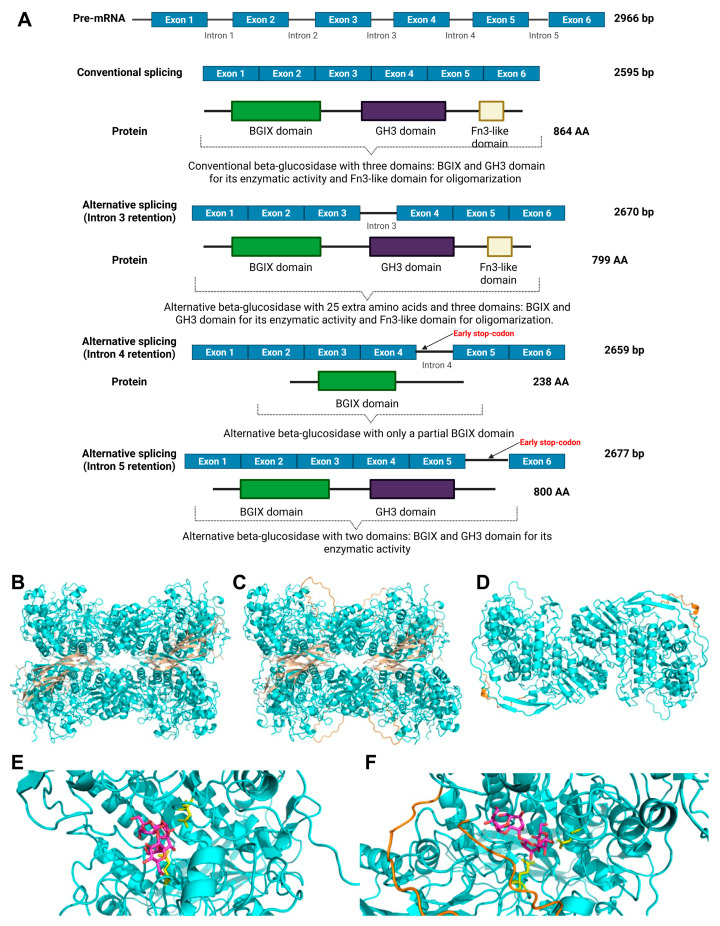
Structural and functional consequences of alternative splicing in the β-glucosidase gene *H101_03896*. (**A**) Schematic representation of the conventionally spliced and intron retention (IR) pre-mRNAs and the different isoforms they generate, including the distinct domain compositions (BGIX, GH3, and Fn3-like domains). (**B**) Structure of the CS H101_03896-encoded protein in its biological assembly (a tetramer). The Bglx and GH3 domains are colored in cyan, while the Fn3-like domain is shown in tan. (**C**) Structure of the biological assembly of the IR3 isoform of H101_03896 (a tetramer). The Bglx and GH3 domains are shown in cyan, the Fn3-like domain is shown in tan, and the added AA are shown in orange. (**D**) Structure of a biological assembly of the IR5 isoform of H101_03896 (a dimer). The Bglx and GH3 domains are shown in cyan, and the added AA are shown in orange. (**E**) The binding mode of a β-glucan tetramer (pink) in the catalytic cleft of the CS H101_03896 protein. The catalytic AAs Asp262 and Glu491 are shown in yellow. (**F**) The binding mode of a β-glucan tetramer (pink) in the catalytic cleft of the IR3 isoform of H101_03896. The catalytic AAs Asp287 and Glu516 are shown in yellow, and the added AA are shown in orange. Created in BioRender. Rossi, A. (2026) https://www.biorender.com/hyg4kr7 (accessed on 4 February 2026).

The β-glucosidase, encoded by *H101_03896*, appears to be a crucial enzyme for *T. interdigitale* adaptation in keratin; *H101_03896* is one of the nine DEGs and ASGs at 24 and 96 h. This enzyme acts on β-glucans in the fungal cell wall. The IR events in this gene were all down-regulated and produced different isoforms (Figure 7A), which may be important for cell wall remodeling because they were all predicted to be extracellular (Supplementary Table S3). The conventionally spliced (CS) and IR3 isoforms form a tetramer, whereas the IR5 isoform forms only a dimer because this IR event introduces an early stop codon, leading to a loss of the Fn3-like domain (Figure 7B–D). The IR3 isoform was present at 24 h, the IR4 isoform appeared at 96 h, and the IR5 isoform, as mentioned, was present at both times.

Next, we conducted a molecular docking analysis with a β-glucan tetramer to determine if the CS and IR3 isoform, which has an additional 25 amino acid residues at the beginning of the protein, interact differently with the substrate. The analysis showed that although the CS isoform could accommodate larger substrates, the IR3 isoform could not, as the additional residues would impede the binding of larger carbohydrates (Figure 7E,F).

### 2.5. Exon Skipping as an Additional Way PacC Regulates Transcription

**Figure 8:**
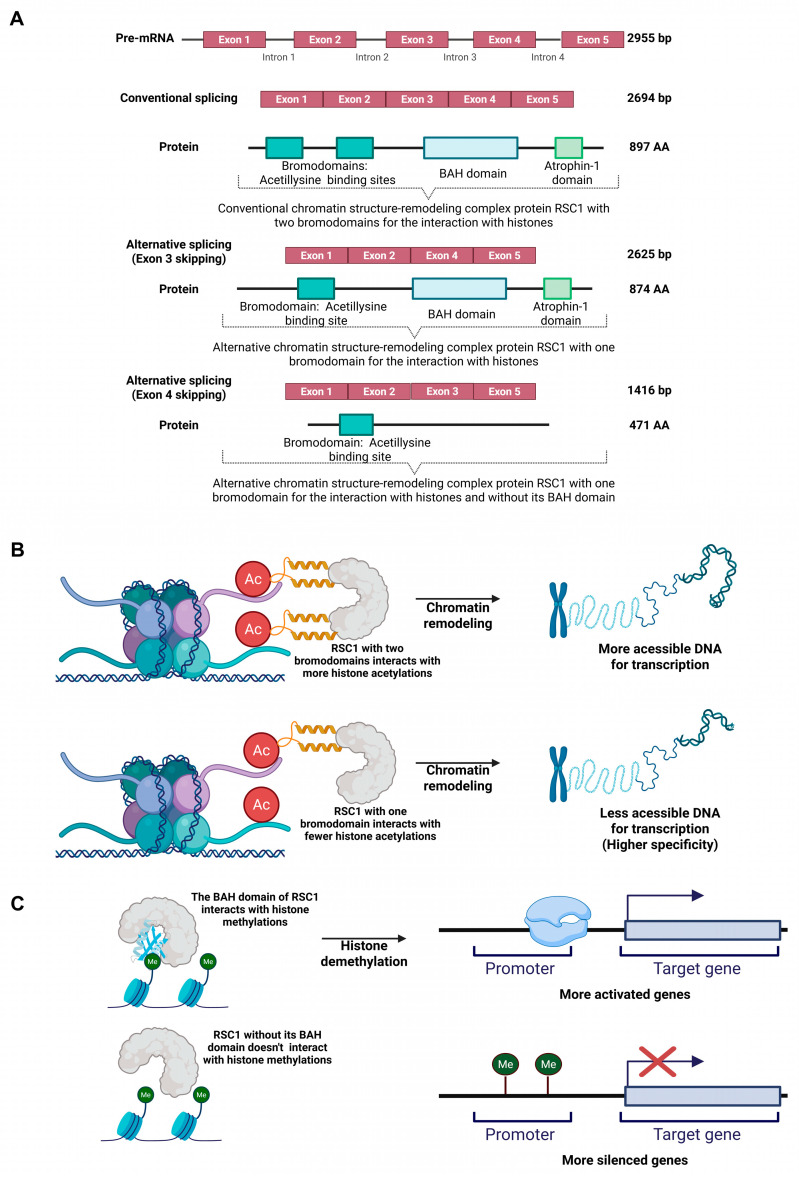
Proposed mechanism of alternative splicing of the chromatin remodeler RSC1 encoded by *H101_00271* generates isoforms with distinct histone-binding capacities and functional outcomes. (**A**) Schematic representation of the RSC1 pre-mRNAs and the protein isoforms produced by conventional and alternative splicing. Top: Conventional splicing produces a transcript containing all five exons, which is translated into the full-length RSC1 protein. This isoform contains two bromodomains, a BAH domain, and an Atrophin-1 domain. Middle: The alternative splicing event that skips exon 3 produces an isoform lacking one bromodomain. Bottom: The alternative splicing event that skips exon 4 produces a shorter isoform lacking the BAH domain and the second bromodomain. (**B**) Schematic of the function of the full-length RSC1 isoform. The presence of two bromodomains allows for interaction with a greater number of acetylated histones, while the BAH domain facilitates binding to methylated histones. This multi-domain engagement promotes widespread chromatin remodeling, leading to more accessible DNA and robust activation of target genes. (**C**) Functional consequences of the alternative splicing events. The isoform with a single bromodomain exhibits reduced capacity to bind acetylated histones, potentially leading to greater promoter specificity. The isoform lacking the BAH domain cannot interact with methylated histones, resulting in altered recruitment and gene activation profiles. These splicing variants may provide a mechanism for fine-tuning chromatin accessibility and transcriptional programs. Created in BioRender. Rossi, A. (2026) https://www.biorender.com/kmnp3ma (accessed on 4 February 2026).

Analysis of the Δ*pacC* mutant showed down-regulation of skipping of exons 3 and 4 in *H101_00271*, which encodes RSC1, a subunit of a chromatin remodeling complex, at both 24 and 96 h. These AS events do not alter the reading frame of the transcripts but produce structurally distinct protein isoforms. However, it is important to note that skipped exon 3 contains one of the two bromodomains of the protein, which interacts with the acetyl-lysine residues in histones, and skipped exon 4 corresponds to the bromo-adjacent homology (BAH) domain, which is involved in histone methylation (Figure 8A). The lack of one acetyl-lysine-binding site in the AS isoform may interfere with chromatin remodeling and thus may directly affect the genes available for transcription (Figure 8B). The lack of a BAH domain may also result in fewer active genes (Figure 8C).

### 2.6. The ΔpacC Mutant Strain Shows Signs of Cytoplasmic Damage and Cell Wall Thinning

**Figure 9:**
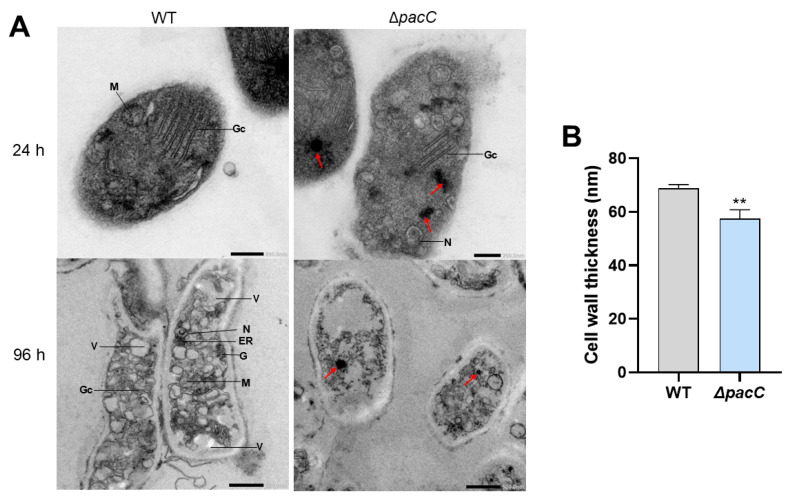
Transmission electron microscopy images of the wild-type and Δ*pacC* mutant strains after 24 and 96 h of growth in keratin. (**A**) Transmission electron microscopy (TEM) of the mycelium of wild-type (WT) and Δ*pacC* strains at 24 and 96 h. Some organelles such as the nucleus (N), endoplasmic reticulum (ER), Golgi complex (Gc), mitochondria (M), glycogen aggregates (G), and vacuole (V) are indicated. Red arrows point to the electron-dense material that may be Woronin bodies. (**B**) Comparison of the cell wall thickness between the WT and Δ*pacC* strains, measured at 10 random points in five hyphae from each strain, analyzed using ImageJ (Version 1.51; Bethesda, MD, USA) Values are the means and standard deviations of the measured points. Data were analyzed using Student’s *t*-test. Significance is indicated as ** *p* < 0.01. Created in BioRender. Rossi, A. (2026) https://www.biorender.com/zc1h6ex (accessed on 4 February 2026).

Transmission electron microscopy (TEM) of WT and Δ*pacC* mutant mycelia grown in keratin (Figure 9A) showed viable hyphae with visible organelles and electron-dense cellular content in both the WT and Δ*pacC* mutant at 24 h. However, one notable difference between the strains is the black spots in the Δ*pacC* mutant (red arrows in Figure 9A). These spots originate from an electron-dense material that may be a Woronin body. At 96 h, the WT strain presented hyphae full of organelles with prominent vacuoles and a thick cell wall. In contrast, the Δ*pacC* mutant had a deranged cytoplasm with no clear organelles and numerous electron-dense spots; the cell wall also appeared to be damaged. We measured the thickness of the cell walls of the WT and Δ*pacC* strains after 96 h of growth in keratin (Figure 9B), which showed that the cell wall of the WT strain was ~17% thicker than that of the Δ*pacC* mutant.

## 3. Discussion

The role of PacC in fungal adaptation to diverse environments [10,27] and its importance in fungal pathogenicity are well documented [8,9,25]. However, the mechanism through which this TF modulates AS remains poorly understood. Our data provide new insights into the role of PacC-mediated AS in *T. interdigitale* infection. We showed that in a Δ*pacC* mutant after 24 and 96 h of growth in infection-mimicking keratin medium, IR was the most common AS event (Figure 1); a phenomenon that has been observed in filamentous and pathogenic fungi [28,29]. Validation of the IR events using qPCR (Figure 5) indicated that the Δ*pacC* mutation may drive a splicing-defective mechanism in several important genes, probably activating the NMD pathway, which is one of the most frequent effects of IR [17]. Two genes affected by the introduction of premature termination codons (PTCs) encode membrane ATPases (*H101_05869* at 24 h and *H101_02524* at 96 h), which are crucial for maintaining the transmembrane electrochemical gradient, membrane potential, and intracellular pH balance [30]. Another important gene that was affected by the probable defective splicing machinery in the Δ*pacC* mutant at both 24 and 96 h encodes a transmembrane calcium transport protein (*H101_00864*). Calcium transport is essential for cell signaling and is involved in fungal growth, virulence, and stress responses [31,32,33]. AS in the mutant triggered PTCs in genes linked to transcriptional regulation and genome maintenance (Supplementary Table S1); these genes are essential for adaptation and stress responses, and their dysfunction can lead to cell death [34,35].

The abnormal IR observed in the Δ*pacC* mutant also affected energy metabolism and biosynthetic pathways (Figure 3). An imbalance in central metabolic reactions, such as those in the TCA cycle and oxidative phosphorylation, disrupts cellular homeostasis and leads to metabolic dysfunction [36]. One of the biosynthetic pathways that was disrupted at 96 h was the ergosterol pathway (Figure 3 and Supplementary Table S2), which is the main sterol in fungal membranes. Ergosterol content impacts membrane fluidity, structure, and permeability [37,38]. An ergosterol quantification assay showed that, after 96 h, the Δ*pacC* mutant had lower ergosterol content than the WT strain (Figure 6). During keratin consumption, dermatophytes secrete ammonia and alkalize the medium [19]. An alkaline pH increases membrane rigidity [39]. To cope with environmental stressors that lead to membrane rigidity, fungi increase ergosterol content [40,41]. Deficiency in ergosterol homeostasis affects viability and stress responses, thereby impairing pathogenicity [41]. These findings show that defective modulation of AS in the Δ*pacC* mutant has extensive effects on fungal metabolism and homeostasis during infection.

Although AS may lead to NMD in pathogenic fungi, it is also linked to virulence and stress responses, which are related to host invasion [29,42]. The down-regulated IR events shown in Figure 5 indicate that PacC modulates AS to produce distinct protein isoforms that may positively affect infection. IR8 of *H101_01659*, encoding a myosin-1, produces a shorter protein missing an SH3 domain, which was shown to be dispensable for myosin-1 activity in the cytoskeleton of *Fusarium graminearum* [43]; IR2 of *H101_05794*, which encodes an AUR protein kinase, removes 18 amino acids at the end of the protein (Supplementary Figure S1). These alternative isoforms may reduce the metabolic burden caused by infection-related stress, as the costs of protein production are high [44,45] and reducing non-essential protein domains would save energy for essential biological processes. IR4 of *H101_04816*, encoding a glutamate carboxypeptidase, results in the loss of a transferrin receptor-like dimer domain (Supplementary Figure S1). Protease dimerization can exert autoinhibitory effects, and removing this domain may yield a more active isoform [46].

In the Δ*pacC* mutant, AS caused IR in the *H101_03896* gene, generating three alternative isoforms of a GH3 β-glucosidase (Figure 7A). These different isoforms may play distinct time-dependent roles in cell wall remodeling, as isoform IR3, present in 24 h, added amino acids that reduce the size of the catalytic cleft of the enzyme (Figure 7F) and isoform IR5, which was present both 24 and 96 h formed a dimer instead of the tetramer formed by the CS and IR3 isoforms (Figure 7D). The cell wall is a key modulator of pathogenesis, and cell wall remodeling is critical for adaptation [47]. The fungal cell wall contains glucans with β-1,3 and β-1,4 linkages that function in resistance (β-1,3-glucans) and organization (β-1,4-glucans) [48]. These linkages are hydrolyzed by β-glucosidases [49,50]. IR4 of the *H101_03896* gene generates a small protein with a partial BGIX domain (Figure 7A). Despite lacking catalytic activity, this protein can bind to glucans and act as an immunological decoy during infection, because glucans frequently function as pathogen-associated molecular patterns [51,52]. Although PacC was previously shown to activate cell-wall remodeling enzymes by stimulating expression of related genes [10,53,54], this is the first report of PacC using modulating AS to achieve this.

IR has been reported as the most common AS event in fungi; however, in our study within 24 h, half of the down-regulated events were ES (Table 2), suggesting that WT *T. interdigitale* may also frequently use ES as a modulation mechanism. The most significant examples are ES3 and ES4 of the gene encoding RSC1, a component of the chromatin structure remodeling complex, at 24 and 96 h (Figure 8A). Chromatin remodelers are epigenetic regulators that can alter transcriptional dynamics. RSC1 is required for fungal pathogenesis and is associated with histone demethylation and nucleosome changes [55]. ES3 in RSCI causes the loss of one bromodomain; this may increase the specificity of the remaining bromodomain, which interacts with histone acetyl-lysine, thereby modulating transcription by reducing accessible DNA (Figure 8B). ES4 in RSCI caused the loss of the BAH domain, leading to reduced histone demethylation and a greater number of silenced genes (Figure 8C). The fungal TF AreB was shown to recruit the chromatin remodeling complex to regulate stress responses [56]. We propose that although ES is not as common as IR in fungi, as ES was previously reported to account for less than 1% of AS events [29], in *T. interdigitale*, PacC can exploit ES to modulate fungal epigenetics via RSC1.

To determine if any of the cellular processes affected by AS dysregulation in the Δ*pacC* mutant grown in keratin could alter fungal physiology, we examined the mycelia using TEM (Figure 9). At both 24 and 96 h, the mycelium of the Δ*pacC* mutant had electron-dense spots, which we hypothesized to be Woronin bodies based on their similarity to the Woronin bodies observed in previous studies [57,58]. These structures seal the pores caused by hyphal wounding and prevent cytoplasmic bleeding [59]. These pores could have been caused by disrupted cell wall remodeling. However, the cellular organelles in the mutant appeared to be healthy at 24 h. However, after 96 h, the cellular content was disrupted, similar to that observed in *T. rubrum* treated with the antifungal terbinafine [60]. Terbinafine directly inhibits ergosterol biosynthesis by targeting the squalene epoxidase ERG1 [61]. The dysregulated AS caused by *pacC* deletion produced nonfunctional isoforms of enzymes involved in ergosterol biosynthesis (Supplementary Table S2), such as C-14 sterol reductase (erg24) and acetoacetyl-CoA reductase (Erg13). The similarity between the Δ*pacC* mutant after 96 h of growth in keratin and other dermatophytes, such as *T. rubrum* treated with terbinafine, could indicate that the non-function isoforms generated by AS are directly related to ergosterol content and the cellular disruption observed at this time point. Cell wall thinning may also be associated with AS. As shown in Figure 7, the WT strain produced different isoforms of β-glucosidase through AS, which could be essential for cell wall remodeling.

**Figure 10:**
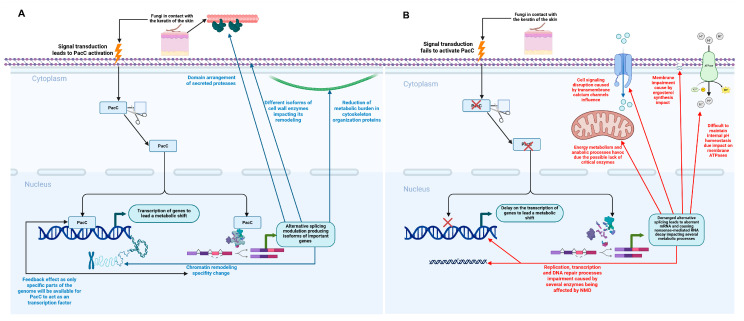
Proposed model of PacC-mediated alternative splicing during infection. (**A**) With a functional PacC, signal transduction leads to activated PacC translocating to the nucleus. As a transcription factor, PacC regulates the AS of genes involved in key processes, such as cell wall enzyme production, cytoskeleton organization, and chromatin modification to drive a metabolic shift and cellular remodeling. (**B**) Without a functional PacC, failure of appropriate AS modulation results in cellular dysregulation. This dysregulation includes delayed gene expression, impaired membrane integrity, disrupted signaling, and defective energy metabolism, all of which are due, in part, to NMD. Red crosses represent the lack of PacC protein.

In conclusion, our findings reveal an extra layer of complexity for the role of PacC in the fungal network during infection, showing that this TF not only governs gene expression in response to ambient pH, but also fine tunes fungal physiology through AS. By regulating both IR and ES, PacC appears to coordinate metabolic balance, membrane stability, cell wall remodeling, and epigenetic regulation, enabling *T. interdigitale* to adapt to the dynamic host environment. Figure 10 shows a proposed model for the role of PacC in modulating AS during infection and the cellular dysregulation resulting from the loss of PacC in harmonizing AS events. Our results broaden the understanding of fungal adaptation strategies, highlighting AS as a pivotal mechanism by which PacC promotes infection success and environmental resilience. Nevertheless, more studies focusing on how PacC interacts with the splicing machinery need to be performed to clarify the molecular mechanisms involved in this phenomenon.

## 4. Materials and Methods

### 4.1. Strains and Growth Conditions

*T. interdigitale* H6 strain (ATCC MYA-3108) was isolated at the University Hospital of Ribeirão Preto Medical School, São Paulo University, Brazil, and the Δ*pacC* strain derived from this strain, which carries a disrupted *pacC* gene, were used in this work [23,25]. These strains were cultured on malt extract agar (pH 5.7) at 28 °C. Conidial suspensions were obtained by flooding 21-day-old plates with sterilized 0.9% NaCl, recovering the liquid, vortexing, and filtering through glass wool. The conidial concentration was estimated by counting in a Neubauer chamber. For growth in liquid medium, 1 × 10^6^ conidia from each strain were inoculated into 50 mL of Sabouraud dextrose broth (SDB; pH 5.7), followed by pre-cultivation at 28 °C for 96 h. After pre-cultivation, the mycelia were transferred to 100 mL of minimal medium (MM) at pH 5.0, as described previously [62], supplemented with keratin (2.5 g/L) as the sole carbon and nitrogen source, and incubated with continuous shaking at 100 rpm for 24 and 96 h.

### 4.2. RNA Extraction and cDNA Synthesis

Total RNA was extracted from mycelia using the Illustra RNAspin Mini Isolation Kit (GE Healthcare, Chicago, IL, USA). RNA concentration and purity were assessed with a NanoDrop ND-100 spectrophotometer (Thermo Fisher Scientific, Waltham, MA, USA). To remove genomic DNA, 400 ng of total RNA was treated with DNase I (Sigma-Aldrich, St. Louis, MO, USA), according to the manufacturer’s protocol. The treated RNA was used for first-strand cDNA synthesis, performed using the Platus Transcriber RNase H-cDNA kit (Sinapse, Miami, FL, USA). The resulting cDNA was quantified, evaluated for purity, and diluted to 70 ng/μL for qPCR analysis.

### 4.3. Alternative Splicing Analysis

To detect AS events in the Δ*pacC* mutant, we conducted RNA-seq on the *T. interdigitale* Δ*pacC* mutant cultivated for 24 and 96 h (Gene Expression Omnibus (GEO) database; accession number: GSE313401).

The RNA-seq reads were aligned to the *T. interdigitale* reference genome using STAR software (version 2.7.11b) [63] and AS events were identified using the ASpli package in R software (version 4.3.1) [64]. Differential expression analysis was performed using the Bioconductor DESeq2 package [65]. Significance was set at a Benjamin–Hochberg adjusted *p*-value of 0.05, and a ±1.0 log_2_ fold change was used as cutoff for significant differences in expression. Genes showing a log_2_-fold change were functionally categorized using Gene Ontology (GO) terms as assigned using the Blast2GO [66].

### 4.4. Quantitative Polymerase Chain Reaction (qPCR)

Independent samples from each time point were used, with triplicate biological and technical replicates. AS events were quantified using Power SYBR™ Green PCR Master Mix (Applied Biosystems, Waltham, MA, USA) with ROX dye as a fluorescent normalizer according to the manufacturer’s instructions. Transcript abundance was quantified using the 2^−ΔΔCt^ method. Forward and reverse primers were used for each reaction (to standardize primer concentration and achieve 90–110% efficiency). The expression levels of *gapdh* and *rpb2* were used as endogenous controls [67]. The primer sequences are listed in Supplementary Table S4. To detect AS events, primers were designed to align either within the retained intron or at the intron-exon junction. The wild-type (WT) strain was used as a control to calculate the abundance of AS transcripts in the Δ*pacC* strain. The results are the fold-change (log_10_) of the mean (standard deviation) in Δ*pacC* vs. the WT with three independent replicates.

### 4.5. Ergosterol Quantification

Ergosterol was quantified as previously described [68]. Briefly, mycelial samples grown in keratin medium for 24 and 96 h were filtered, and their dry weights were measured. The dried samples were treated with 3 mL of 25% alcoholic potassium hydroxide solution and vortexed for 5 min. Then, the mixture was incubated at 85 °C for 1 h to complete saponification. After cooling to room temperature, the solution was partitioned by adding 1 mL of sterile distilled water and 3 mL of n-heptane, followed by vigorous vortexing for 5 min. The organic (n-heptane) layer was then carefully transferred to a new polypropylene tube. A 20 µL aliquot of this extract was diluted five-fold in 100% ethanol, and its absorbance was measured from 230 to 300 nm using a spectrophotometer. The ergosterol concentration was determined by interpolating the spectrophotometric data against a standard calibration curve constructed using purified ergosterol (Sigma-Aldrich). Independent samples from each time point were used, with triplicate biological and technical replicates.

### 4.6. In Silico Analyses

Data from Ensembl fungi were used to predict the transcripts and proteins generated from conventional and alternative splicing (https://fungi.ensembl.org/index.html, accessed on 11 June 2025). Reading frames and domains were predicted using the Expasy Translate tool (https://web.expasy.org/translate/, accessed on 11 June 2025) and Conserved Domain Database (https://www.ncbi.nlm.nih.gov/Structure/cdd/wrpsb.cgi, accessed on 11 June 2025), respectively. Subcellular localization was determined using DeepLoc 2.0 (https://services.healthtech.dtu.dk/services/DeepLoc-2.0/, accessed on 23 August 2025). Graphical representations of the isoforms and the resulting proteins were generated using BioRender.

### 4.7. Three-Dimensional Modelling and Molecular Docking

Structural 3D modelling was performed using the AlphaFold web server (alphafoldserver.com). The stereochemical quality of the modeled structures was evaluated using PROSESS [69]. Biological assemblies were generated using the GalaxyWeb server and GalaxyDimer tool (https://galaxy.seoklab.org/). The β-1,4-glucan tetramer was generated using its SMILES code with the NovoPro tool (https://www.novoprolabs.com/tools/smiles2pdb (accessed on 25 June 2025)), and molecular docking calculations were performed using DOCKTHOR (https://dockthor.lncc.br/v2/index.php (accessed on 25 June 2025)). The search space for the docking calculations was defined by a 20 Å × 20 Å × 20 Å cube centered on the conserved catalytic cleft of GH3 enzymes. The exhaustiveness was set to 10, and the default values were used for all other parameters. Three-dimensional molecular models were visualized using the PyMOL Molecular Graphics System (version 2.0; Schrödinger, LLC, New York, NY, USA).

### 4.8. Transmission Electron Microscopy

Mycelial samples from the Δ*pacC* mutant and WT *T. interdigitale* H6 strain cultivated in keratin for 24 and 96 h were fixed in a paraformaldehyde-glutaraldehyde solution for 24 h and post-fixed with 1% osmium tetroxide in sodium cacodylate buffer (0.1 M; pH 7.2). The samples were then dehydrated in graded ethanol and embedded in epoxy resin at 60 °C for 48 h. After washing with propylene oxide, the samples were cut into ultrathin sections and analyzed using a JEOL JEM-100CXII (Akishima, Japan) electron microscope and a Hamamatsu ORCA-HR digital camera (Hamamatsu City, Japan).

### 4.9. Statistical Analyses

Statistical analyses were conducted using an unpaired *t*-test, and significance was determined using the Holm–Sidak method. Significance is indicated as follows: * *p* < 0.05, ** *p* < 0.01, and *** *p* < 0.001. Graphs and statistical analyses were performed using GraphPad Prism Software (version 8.4.3; San Diego, CA, USA).

## Figures and Tables

**Table 1:** Description of the up-regulated alternative splicing events in the Δ*pacC* mutant vs. the wild-type strain.

Time (h)	Intron 1 Retention	Intron 2 Retention	Others	Total
24	128	126	181	435
96	261	300	401	961

**Table 2:** Description of the down-regulated alternative splicing events in the Δ*pacC* mutant vs. the wild-type strain.

Time (h)	Exon Skipping	Intron Retention	Total
24	11	11	22
96	16	79	95

## Data Availability

The original contributions presented in this study are included in the article/Supplementary Materials. Further inquiries can be directed to the corresponding author.

## Supplementary Materials

The following supporting information can be downloaded at: https://www.mdpi.com/article/10.3390/ijms27062634/s1.

## Abbreviations

The following abbreviations were used in this manuscript:

AS	Alternative splicing
IR	Intron retention
NMD	Nonsense-mediated RNA decay
WT	Wild type
