# Supplementary material for: Alternative Splicing Analysis Revealed That the Transcription Factor PacC Shapes the Virulence of the Dermatophyte Trichophyton interdigitale
Source: Int J Mol Sci. 2026 Mar 13;27(6):2634. doi: 10.3390/ijms27062634 (PMC13026334; doi:10.3390/ijms27062634)
Supplement: Supplementary file 1 [file ijms-27-02634-s001.zip › Supplementary material.pdf]

Alternative Splicing Analysis Revealed That the Transcription Factor PacC Shapes the Virulence of the Dermatophyte *Trichophyton interdigitale*

Table S1. Alternative Splicing Landscape of Genes linked to Transcriptional Regulation and Genome Maintenance in the Δ*pacC* mutant vs. the wild-type strain

Gene ID	AS event	Time point (h)	Description	Fold change
H101_05712	Intron 3 retention	24	GATA transcription factor	3,93
H101_04614	Intron 1 retention	24	replication factor C subunit 5	2,62
H101_05711	Intron 2 retention	24	ssDNA binding protein Ssb3	2,45
H101_06306	Intron 1 retention	24	DEAD/DEAH box RNA helicase	2,26
H101_05364	Intron 5 retention	24	mRNA cleavage factor complex component Pcf11	2,09
H101_01850	Intron 2 retention	24	SNF2-family ATP dependent chromatin remodeling factor snf21	2,05
H101_06488	Intron 1 retention	24	replication protein A DNA-binding subunit	2,01
H101_01229	Intron 7 retention	24	mRNA cleavage and polyadenylation specificity factor complex subunit Pta1	1,84
H101_02678	Intron 2 retention	24	RNA-binding protein Nab3	1,76
H101_00068	Intron 3 retention	24	pre-mRNA-splicing factor syf1	1,72
H101_05274	Intron 2 retention	24	SWI-SNF complex subunit	1,74
H101_02474	Intron 1 retention	24	C6 transcription factor	1,62
H101_01615	Intron 1 retention	24	SYF2 splicing factor family protein	1,59
H101_06981	Intron 1 retention	24	small nuclear ribonucleoprotein-associated protein B	1,52
H101_00068	Intron 1 retention	24	pre-mRNA-splicing factor syf1	1,46
H101_08017	Intron 2 retention	24	MYB DNA-binding domain containing protein	1,43
H101_06437	Exon 10 skipping	24	DNA repair protein Rad18	1,42
H101_02736	Intron 5 retention	24	DNA polymerase delta subunit Cdc27	1,41
H101_07863	Intron 5 retention	24	SNF2 family helicase	1,29
H101_02262	Intron 2 retention	24	C6 transcription factor	1,29
H101_00139	Intron 1 retention	24	chromosome segregation protein	1,25
H101_08184	Intron 2 retention	24	DNA-directed RNA polymerase I and III polypeptide	1,23

H101_00947	Intron 1 retention	24	C6 transcription factor	1,18
H101_06392	Intron 3 retention	24	transcription regulator BDF1	1,18
H101_07863	Intron 4 retention	24	SNF2 family helicase	1,18
H101_03467	Intron 1 retention	24	mRNA-nucleus export ATPase	1,12
H101_03712	Intron 2 retention	24	DNA repair protein rhp51	1,09
H101_03578	Intron 3 retention	24	C6 transcription factor	1,09
H101_02736	Intron 7 retention	24	DNA polymerase delta subunit Cdc27	1,06
H101_02474	Intron 3 retention	24	C6 transcription factor	1,06
H101_07863	Exon 6 skipping	24	SNF2 family helicase	1,01
H101_07863	Exon 5 skipping	24	SNF2 family helicase	1,00
H101_00271	Exon 4 skipping	24	chromatin structure-remodeling complex protein RSC1	-1,36
H101_00271	Exon 3 skipping	24	chromatin structure-remodeling complex protein RSC1	-1,40
H101_05531	Intron 1 retention	96	C6 transcription factor	4,31
H101_02549	Intron 14 retention	96	DNA mismatch repair protein msh5	4,20
H101_02549	Intron 11 retention	96	DNA mismatch repair protein msh5	4,11
H101_02549	Intron 10 retention	96	DNA mismatch repair protein msh5	3,91
H101_04262	Intron 1 retention	96	DNA topoisomerase 2	3,80
H101_02549	Intron 9 retention	96	DNA mismatch repair protein msh5	3,78
H101_03082	Intron 3 retention	96	Ku family DNA helicase	3,67
H101_05988	Intron 1 retention	96	C6 transcription factor	3,54
H101_04050	Intron 2 retention	96	pumilio-family RNA binding protein	3,44
H101_04050	Intron 6 retention	96	pumilio-family RNA binding protein	3,36
H101_01070	Intron 1 retention	96	pre-mRNA splicing factor	3,00
H101_03708	Intron 3 retention	96	chromosome segregation protein Spc105	2,93
H101_06306	Intron 1 retention	96	DEAD/DEAH box RNA helicase	2,91
H101_02549	Intron 15 retention	96	DNA mismatch repair protein msh5	2,83
H101_03925	Intron 2 retention	96	transcription factor SipA3	2,81

H101_00491	Intron 1 retention	96	U5 small nuclear ribonucleoprotein component	2,66
H101_02078	Intron 2 retention	96	DNA polymerase epsilon catalytic subunit A	2,61
H101_07989	Intron 2 retention	96	RNA-binding post-transcriptional regulator csx1	2,58
H101_02549	Intron 16 retention	96	DNA mismatch repair protein msh5	2,53
H101_03199	Intron 2 retention	96	Rad2-like endonuclease	2,47
H101_03732	Intron 2 retention	96	transcription initiation factor TFIId 127kD subunit	2,33
H101_02009	Intron 2 retention	96	ATP-dependent DNA helicase	2,33
H101_00491	Intron 3 retention	96	U5 small nuclear ribonucleoprotein component	2,26
H101_00453	Intron 2 retention	96	DNA repair/transcription protein	2,25
H101_02474	Intron 3 retention	96	C6 transcription factor	2,19
H101_02419	Intron 1 retention	96	chromatin remodeling complex subunit Arp8	2,17
H101_01689	Intron 5 retention	96	ATP-dependent RNA helicase DOB1	2,14
H101_00453	Intron 3 retention	96	DNA repair/transcription protein	2,13
H101_03732	Intron 3 retention	96	transcription initiation factor TFIId 127kD subunit	2,06
H101_06392	Intron 3 retention	96	transcription regulator BDF1	2,06
H101_02474	Intron 2 retention	96	C6 transcription factor	2,03
H101_01521	Intron 2 retention	96	transcription elongation factor spt6	2,01
H101_05191	Intron 1 retention	96	transcription factor TFIIA complex subunit Toa1	1,96
H101_02975	Intron 1 retention	96	SAGA complex component Sgf29	1,95
H101_02186	Intron 1 retention	96	C6 transcription factor	1,89
H101_05679	Intron 2 retention	96	C6 transcription factor	1,89
H101_05641	Intron 1 retention	96	RNA polymerase II mediator complex protein Nut2	1,89
H101_04540	Intron 1 retention	96	Transcription elongation factor elf1-like protein	1,80
H101_03636	Intron 1 retention	96	chromatin regulatory protein sir2	1,79
H101_03527	Intron 1 retention	96	SNF2 family helicase/ATPase	1,68
H101_00155	Intron 2 retention	96	transcription factor TFIID complex 145 kDa subunit	1,62

H101_02942	Intron 1 retention	96	General negative regulator of transcription subunit 4	1,61
H101_06626	Intron 3 retention	96	DNA topoisomerase III	1,58
H101_00947	Intron 1 retention	96	C6 transcription factor	1,58
H101_07301	Intron 4 retention	96	U6 snRNA-associated Sm-like protein LSm5	1,57
H101_04052	Intron 1 retention	96	RNA polymerase TFIIH complex subunit Ssl1	1,55
H101_04581	Intron 2 retention	96	C6 transcription factor	1,55
H101_06759	Intron 2 retention	96	DNA-directed RNA polymerase III subunit RPC3	1,54
H101_01481	Intron 2 retention	96	zinc finger transcription factor	1,53
H101_04520	Intron 1 retention	96	nitrogen assimilation transcription factor nirA	1,52
H101_01670	Intron 2 retention	96	C6 transcription factor	1,51
H101_05364	Intron 5 retention	96	mRNA cleavage factor complex component Pcf11	1,47
H101_06009	Intron 4 retention	96	C6 transcription factor	1,47
H101_03147	Intron 2 retention	96	AT DNA binding protein	1,42
H101_06966	Intron 1 retention	96	forkhead transcription factor	1,41
H101_05542	Intron 2 retention	96	bZIP transcription factor	1,41
H101_02474	Intron 1 retention	96	C6 transcription factor	1,40
H101_00145	Intron 2 retention	96	fungal specific transcription factor domain-containing protein	1,39
H101_04520	Intron 3 retention	96	nitrogen assimilation transcription factor nirA	1,34
H101_06437	Exon 10 skipping	96	DNA repair protein Rad18	1,30
H101_05890	Intron 5 retention	96	RNA binding domain-containing protein	1,29
H101_02549	Intron 5 retention	96	DNA mismatch repair protein msh5	1,28
H101_03578	Intron 3 retention	96	C6 transcription factor	1,28
H101_03978	Intron 2 retention	96	RNA binding domain-containing protein	1,26
H101_02453	Intron 1 retention	96	TFIIIC transcription initiation factor complex subunits Tfc3	1,25
H101_06406	Intron 2 retention	96	CCCH finger DNA binding protein	1,25
H101_00947	Intron 2 retention	96	C6 transcription factor	1,25
H101_00195	Intron 1 retention	96	C6 transcription factor Ctf1A	1,17

H101_04381	Intron 2 retention	96	pre-mRNA-splicing factor cwc15	1,16
H101_05070	Intron 1 retention	96	C2H2 transcription factor	1,16
H101_04307	Intron 2 retention	96	Poly(A) polymerase PAPalpha	1,15
H101_04945	Intron 2 retention	96	RNase H domain-containing protein	1,15
H101_03578	Intron 2 retention	96	C6 transcription factor	1,13
H101_02695	Intron 3 retention	96	C6 transcription factor	1,13
H101_04520	Intron 2 retention	96	nitrogen assimilation transcription factor nirA	1,13
H101_00524	Intron 1 retention	96	transcription initiation factor IIA gamma chain	1,13
H101_05303	Intron 2 retention	96	pre-mRNA splicing factor	1,12
H101_01494	Intron 1 retention	96	transcription factor RfeF	1,12
H101_04540	Intron 2 retention	96	Transcription elongation factor elf1-like protein	1,11
H101_00302	Intron 2 retention	96	DNA-binding protein	1,10
H101_00195	Intron 6 retention	96	C6 transcription factor Ctf1A	1,09
H101_07533	Intron 4 retention	96	C6 transcription factor	1,08
H101_03923	Intron 1 retention	96	SAGA complex subunit Ada2	1,07
H101_06833	Intron 1 retention	96	nuclear transcription factor Y subunit B-7	1,04
H101_06833	Intron 3 retention	96	nuclear transcription factor Y subunit B-7	1,02
H101_01673	Intron 2 retention	96	transcription factor TFIIIB complex subunit brf1	1,02
H101_00271	Exon 4 skipping	96	chromatin structure-remodeling complex protein RSC1	-1,34
H101_00271	Exon 3 skipping	96	chromatin structure-remodeling complex protein RSC1	-1,49
H101_06437	Intron 6 retention	96	DNA repair protein Rad18	-2,15

Table S2. Description of AS events in genes involved in the ergosterol biosynthesis pathway occurring in the Δ*pacC* mutant vs. the wild-type strain after 96 h of growth in keratin.

Gene ID	Description	AS event	Size of conventional protein isoform	Size of alternative protein isoform
H101_06228	C-14 sterol reductase	Intron 1 retention	499	36
H101_04485	sterol O-acyltransferase	Intron 1 retention	542	43
H101_00192	acetoacetyl-CoA reductase	Intron 1 retention	905	60
H101_06228	C-14 sterol reductase	Intron 2 retention	499	143
H101_06869	isopentenyl-diphosphate delta-isomerase	Intron 2 retention	267	210
H101_00287	oxidosqualene:lanosterol cyclase	Intron 3 retention	717	688
H101_04485	sterol O-acyltransferase	Intron 4 retention	542	526
H101_00192	acetoacetyl-CoA reductase	Intron 5 retention	905	322

Table S3. Subcellular localization prediction for the protein encoded by gene H101_03896 (β-glucosidase) and its alternative splicing isoforms

Localization	H101_03896 conventional isoform	H101_03896 intron 3 retention	H101_03896 intron 4 retention	H101_03896 intron 5 retention
	Probability			
Cytoplasm	0.1775	0.1939	0.1142	0.1337
Nucleus	0.0839	0.0958	0.0247	0.0541
Extracellular	0.6727	0.6508	0.8086	0.7015
Cell membrane	0.1273	0.1273	0.0922	0.1128
Mitochondrion	0.0756	0.0725	0.0741	0.0841
Plastid	0.0075	0.0068	0.003	0.0075
Endoplasmic reticulum	0.3619	0.3513	0.3018	0.3238
Lysosome/Vacuole	0.4938	0.469	0.3555	0.4779
Golgi apparatus	0.1197	0.1211	0.1141	0.1193
Peroxisome	0.0022	0.0029	0.0007	0.0006

Generated using the DeepLoc 2.0 server. The table shows the predicted localization probabilities for the conventional isoform and three intron-retention events (intron 3, intron 4, and intron 5). Higher probability values indicate a greater likelihood of the protein being localized in the respective cellular compartment.

Table S4. List of primers used in this study

Gene ID	Event	Primer Forward	Primer Reverse
H101_03896	Intron 3 Retention	TACTGCACGTTTGCCTCCAT	GTAGGCTAGTTCTCTGCTCCA
H101_03896	Intron 4 Retention	GAAGCTCAGGGGTATGGAT	CCATCCGAAAGTCATCATC
H101_03896	Intron 5 Retention	CCTGTTCTCCCCGCTAGT	GATAGGAGGAAGCAAAGAAAGG
H101_00864	Intron 5 Retention	ATACCCCCAATTCATCCAGG	ATGTCGGTGTCGTTGACA
H101_05869	Intron 2 Retention	GTACGGTCTCAACCAGATGA	AGCTTGCTTACCTCCATG
H101_01659	Intron 8 Retention	GGCGATGTGTATGTTTCACC	GTTGAAGTCGATCCTAAGCTCG
H101_02524	Intron 1 Retention	CACAGAAACGGATTGCTG	GCTTGCCAGTCCTGATAA
H101_04816	Intron 4 Retention	GAAGGTTATCTGGCCGTAC	GGCAACTTACCGATGCTAT
H101_05794	Intron 2 Retention	GGAGAGATGACCATCCCAT	CTGGACGTACCCATACAG

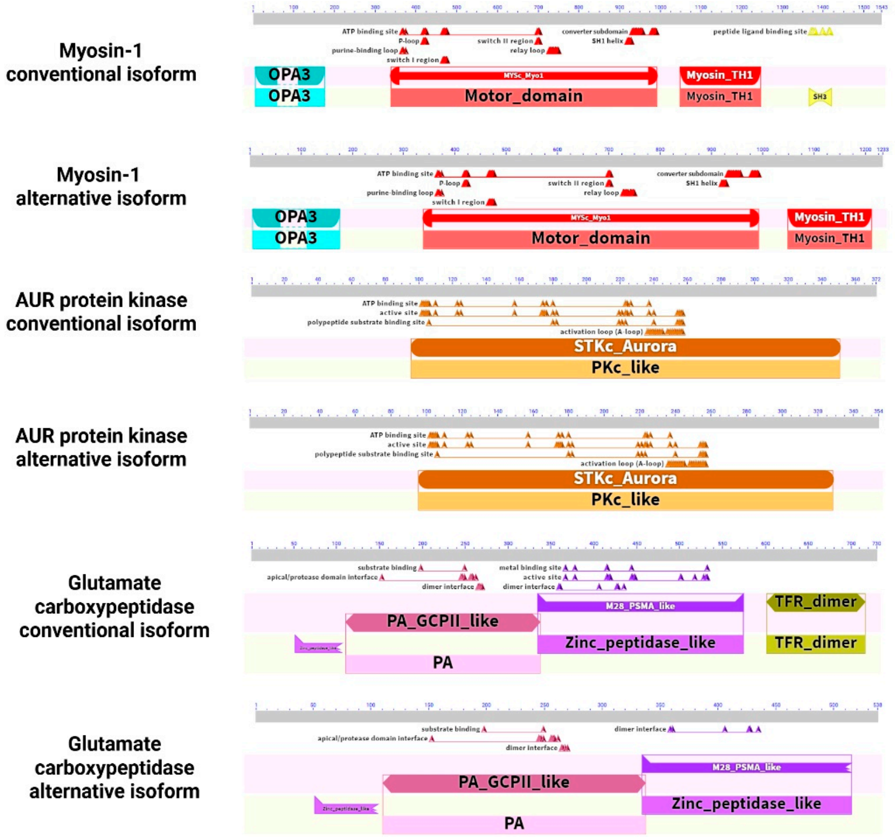

Figure S1. Comparison of conventional and alternative isoforms of the proteins generated from genes H101_01659, H101_05794, and H101_04816.
